# Improving detection of candidates for botulinum toxin therapy in spasticity: an interprofessional screening approach

**DOI:** 10.3389/fneur.2026.1939701

**Published:** 2026-10-15

**Authors:** Melanie Rudelstorfer, Brigitte Mayr-Pirker, Eugen Trinka, Bernhard Iglseder, Richard Friedrich Radlberger

**Affiliations:** 1 Department for Orthopedics and Traumatology, University Hospital Salzburg, Paracelsus Medical University, Salzburg, Austria; 2 Nursing Management, University Hospital Salzburg, Paracelsus Medical University, Salzburg, Austria; 3 Department of Geriatric Medicine, University Hospital Salzburg, Paracelsus Medical University, Salzburg, Austria; 4 Institute of Palliative Care, Paracelsus Medical University, Salzburg, Austria; 5 Department of Neurology, Neurological Intensive Care and Neurorehabilitation, Christian Doppler Medical Centre, Paracelsus Medical University, Salzburg, Austria; 6 Neuroscience Institute, Christian Doppler University Hospital, Centre of Cognitive Neuroscience, Paracelsus Medical University, Salzburg, Austria; 7 Karl Landsteiner Institute for Clinical Neuroscience, Salzburg, Austria; 8 Institute of Public Health, Medical Decision-Making and HTA, UMIT - Private University for Health Sciences, Medical Informatics and Technology, Hall in Tyrol, Austria

**Keywords:** botulinum toxin, diagnostic accuracy, interprofessional screening approach, nurses, occupational therapy, physiotherpy, spastic movement disorders

## Abstract

**Background:**

Spastic movement disorders (SMD) are commonly treated with botulinum toxin, however, many patients who may benefit from treatment remain unrecognized in routine clinical practice. Early identification is particularly challenging in non-specialized settings. This study evaluated the diagnostic performance of interprofessional screening tools for identifying patients with clinically relevant SMD requiring referral for specialist assessment.

**Methods:**

In this prospective, two-phase, single-center study, 124 patients who received care by departments of neurology and geriatrics were included. In phase 1, the Spastic Movement Disorder decision tree (SMD-DT) was applied independently by nurses, physiotherapists, and occupational therapists and compared with the clinical assessment of an experienced neurologist as the reference standard. Based on the findings, a simplified nursing screening tool, the Salzburg Nursing Questionnaire (SNQ), was developed and subsequently evaluated in independent cohorts in phase 2 of the study.

**Results:**

The SMD-DT demonstrated high agreement with the reference standard in neurological rehabilitation settings, with a high degree of consistency across all professional groups compared with the expert, but showed substantially lower performance among nurses in non-specialized geriatric settings. In contrast, the SNQ consistently achieved high diagnostic accuracy in these settings (sensitivity of 1.00 and specificity of 0.96) considering treatment recommendation.

**Conclusion:**

The diagnostic performance of screening instruments for SMD depends strongly on both clinical context and professional background. These findings support the implementation of profession-specific screening strategies to improve early recognition of clinically relevant SMD, facilitating timely referral for specialist evaluation, and optimize access to botulinum toxin therapy in routine clinical practice.

## 1 Introduction

Spastic movement disorders (SMD) are a common consequence of neurological diseases and are associated with pain, long-term motor impairment and reduced quality of life (1, 2). Stroke is one of the leading causes of persistent functional impairment in adults worldwide and represents a substantial burden for both patients and healthcare systems (3). Among sequelae, post-stroke spasticity (PSS) is a common and clinically relevant complication that emerges during motor recovery. Its prevalence is dynamic, ranging from 4 to 42.6%, depending on the timing of assessments (1, 2). Patients presenting with severe initial upper limb paresis are at particularly high risk, with up to 46% developing spastic hypertonia within the first year after stroke (4).

Despite the availability of effective and guideline-recommended treatment, a substantial proportion of patients who may benefit from botulinum toxin therapy are never referred for specialist assessment (5–7). This gap is especially pronounced in geriatric and multimorbid populations, where competing medical priorities may obscure the functional impact of spasticity and contribute to under-recognition and under-referral (5, 8, 9).

Delayed or missed identification remains a critical barrier to optimal care, particularly in non-specialized settings where structured neurological assessments are less consistently implemented. Yet timely detection is essential to prevent secondary complications including contractures, pain syndromes and sustained limitations in activities of daily living (10).

Optimal management of SMD requires an interdisciplinary and interprofessional approach integrating neurology, neurosurgery and rehabilitation medicine, as well as physiotherapy, occupational therapy, orthotics and nursing care. Within this framework botulinum toxin constitutes a cornerstone of focal therapeutic strategies (7). However, timely access to such specialized interventions depends on recognition and appropriate referral pathways.

Given the limited time physicians spend at the bedside – particularly in the longitudinal assessment of evolving spasticity – there is a clear need for pragmatic and scalable screening approaches. Structured tools capable of identifying patients at risk and facilitating referral to specialized care may help bridge the gap between prevalence and treatment. To address this need, established scales – the Modified Ashworth Scale (MAS) (11) and the Medical Research Council (MRC) scale (12), routinely used by physiotherapists and occupational therapists – were combined with the Spasticity-Associated Arm Pain Scale (SAAPS) (13) within the “Spastic Movement Disorder Decision Tree (SMD-DT)” (14). The composite approach aims to stratify patients according to clinically relevant spasticity and potential treatment benefit (10).

In this context, the present study evaluates whether the implementation of a structured screening instrument can improve the detection of clinically relevant spasticity and enhance referral to appropriate treatment pathways.

## 2 Methods

### 2.1 Setting and participants

This prospective two-phase single-centre study enrolled 124 patients between April and October 2025. All of them were recruited at the University Departments of Neurologys’ and of Geriatrics’ wards as well as in the affiliated long-term care facility of the Christian Doppler University Hospital. Accordingly, the respective indications for admission were acute neurological conditions, remobilisation in older adults (>65 years of age), or severe neurological deficits with a permanent, high level of care dependency.

### 2.2 Study design

This study was executed in two clinical phases to evaluate and optimize the identification of spasticity in patients with neurological disorders using both, the SMD-DT (14) and a newly developed nursing assessment instrument. At all times, each profession was blinded to the assessments made by the others. It was ensured that no invasive or burdensome procedures were performed.

Initially, the SMD-DT was evaluated on a geriatric ward, where referrals for botulinum toxin therapy were relatively infrequent. It was implemented by physiotherapists, occupational therapists and nursing staff, as recommended in the initial publication (14). A preliminary pilot study revealed that, despite brief training, nursing staff experienced difficulties applying the decision tree in this setting. Subsequently, the use of the SMD-DT was evaluated on a neurological rehabilitation unit (A), where patients presented with more complex conditions and staff were more frequently involved in the management of spasticity.

Based on insights gained from the first phase, an interprofessional meeting took place between the nursing staff and an experienced neurologist, well versed in the use of botulinum toxin. Within this discussion three main issues with SMD, from a caregiver perspective, were singled out by consensus to be included into the nursing assessment instrument “Salzburg Nursing Questionnaire” (SNQ) (Table 1) as pragmatic approach to facilitate the early detection of spasticity. The questionnaire is suggested to be filled out after the morning care routine (administering medication, washing and dressing) and results in a referral recommendation if any of the questions are answered with ‘yes’ based on a SMD.

**Table 1:** Salzburg nursing questionnaire.

Salzburg nursing questionnaire (SNQ)		
Nursing indication spasticity		
1.	Do I require physical strength during personal care due to the patient’s tense posture? e.g. genital area, armpits, urinary catheter change	O
2.	Is pain perceived during mobilization? e.g. during dressing, personal hygiene	O
3.	Are there skin injuries (macerations/ulcerations) due to the tense posture? e.g. palms, elbows, armpits	O

English forward translation of the Salzburg Nursing Questionnaire (SNQ). The study was conducted using the original German questionnaire. The English version is an author-generated forward translation provided for international readers and has not been formally validated. The original German questionnaire is included in the Supplementary material.

In the second phase of the study the referral recommendation by the SNQ used by the nursing staff and SMD-DT used by the physician was compared on an independent neurological rehabilitation unit (B) to minimize potential training effects. After the questionnaire proved suitable, it was subsequently tested in a nursing home specializing in multiple sclerosis and vegetative state.

### 2.3 Training and implementation

Prior to implementation, all participating nursing staff in phase 1 and all therapy staff in either phase received structured in person training as presentation and Q&A session with the neurologist of roughly 45 min as well as beforehand access to the online available training videos (15) on the application of the scales used in the SMD-DT. Hereinafter, before the SNQ was used in phase 2, SMD followed by the questionnaire itself, instead of the scales used in the SMD-DT, was reviewed with participating nursing staff as part of a presentation followed by a Q&A session with the neurologist of roughly 30 min. The aim of this training was to improve the detection of spasticity and to ensure standardized use of the instruments, thereby enabling timely referral for specialized treatment. Furthermore, to avoid learning effects, no staff members other than the physician were involved in both phases.

### 2.4 Statistical analysis

Descriptive statistics were performed for demographic and etiological data. The inter-rater reliability compared to the SMD-DT assessment of the neurologist, as gold standard, was done by calculating Cohens kappa (κ) with the exact method and 95% confidence intervals were computed based on simple bootstrapping with 1,000 resamples by IBM SPSS Statistics (16). Diagnostic performance of the screening tools was evaluated by calculating the sensitivity and specificity considering a referral recommendation resulted in a later usage of botulinum toxin. Here the neurologist conducted the clinical examination and determined the indication for mentioned botulinum toxin therapy. Confidence intervals for sensitivity and specificity were calculated by Clopper-Pearson confidence intervals using MedCalc (17). *p*-values of less than 0.05 were considered significant.

## 3 Results

A total of 124 participants (56 in the geriatrics’ ward; 12 in rehabilitation unit (A); 28 in rehabilitation unit (B); 28 in the long-term care centre) were included in the analysis. The median age was 72 years with a range of 20 to 94 years and 57 participants (46%) were male. The most common main diagnoses at admission were ischemic or haemorrhagic stroke (*n* = 41; 33.1%), followed by traumatic aetiologies such as fractures of the lower extremities or spine, traumatic brain injury, and paraplegia (*n* = 38; 30.6%). Neurodegenerative disorders accounted for only a small proportion (4.8%; *n* = 6). In contrast, other conditions – including viral infections, internal medicine diseases, and multiple sclerosis – were similarly frequent, comprising 31.5% (*n* = 39) of cases (Table 2).

**Table 2:** Basic data of participants.

Characteristics	*n* = 56	*n* = 12	*n* = 28	*n* = 28	*n* = 124
Ward	G	A	B	C	∑
Male, *n* (%)	19 (34)	6 (50)	16 (57)	16 (57)	57 (46)
Age years; median (range)	83 (64–94)	67 (20–75)	62 (32–82)	52 (26–76)	72 (20–94)
Ischemic or hemorrhagic stroke, *n* (%)	13 (23.2)	6 (50)	17 (60.7)	5 (18.5)	41 (33.1)
Trauma (fractures of the lower extremities and spine, traumatic brain injury, paraplegia), *n* (%)	22 (39.3)	3 (25.0)	6 (21.4)	7 (25.9)	38 (30.6)
Neurodegenerative (dementia, parkinson’s disease), *n* (%)	5 (8.9)	0 (0.0)	1 (3.6)	0 (0.0)	6 (4.8)
Other (viral infections, internal medicine diseases, multiple sclerosis), *n* (%)	16 (28.6)	3 (25.0)	4 (14.3)	16 (59.3)	39 (31.5)

Demographic and clinical characteristics of the study population (*n* = 124) divided by ward and for the whole group. Data are presented as number (percentage) or mean (standard deviation), including age distribution and underlying diagnostic categories.

A = rehabilitation unit (A), B = rehabilitation unit (B), C = Long-term care facility, G = geriatrics war, ∑ = whole cohort.

### 3.1 Phase 1

In the geriatric ward, agreement with the SMD-DT by the physician as the reference standard, ranged from fair to substantial (18) across professional groups (Table 3). The highest agreement was observed for occupational therapists (κ = 0.65; 95% CI: −0.03-1.00; *p* = 0.006), followed by the physiotherapists (κ = 0.46; 95% CI: −0.05-0.85; *p* = 0.02), whereas nurses demonstrated lower agreement (κ = 0.28; 95% CI: −0.06-0.63; *p* = 0.069). Sensitivity was identical across professions (0.67), while specificity varied, ranging from 0.87 (nurses) to 0.98 (occupational therapists).

**Table 3:** Phase 1.

Phase 1								
Geriatric ward								
vs. Physician SMD-DT	Cohen’s ϰ	*ρ*-value	95% CI	*n*	sensitivity	95% CI	specificity	95% CI
Physiotherapist	0.46	0.020	[−0.05, 0.85]	54	0.67	[9.43, 99.16]	0.94	[83.76, 98.77]
Occupational therapist	0.65	0.006	[−0.03, 1.00]	56	0.67	[9.43, 99.16]	0.98	[89.93, 99.95]
Nurse SMD-DT	0.28	0.069	[−0.06, 0.63]	56	0.67	[9.43, 99.16]	0.87	[74.66, 94.52]
Physician SMD-DT	-	-	-	56	1.00	[29.24, 100.00]	1.00	[93.28, 100.00]

Neurological rehabilitation Unit A								
vs. Physician SMD-DT	Cohen’s ϰ	*ρ*-value	95% CI	*n*	sensitivity	95% CI	specificity	95% CI
Physiotherapist	1.00	0.003	[1.00, 1.00]	11	0.80	[28.36, 99.49]	1.00	[54.07, 100.00]
Occupational therapist	0.79	0.024	[0.00, 1.00]	11	0.60	[14.66, 94.73]	1.00	[54.07, 100.00]
Nurse SMD-DT	1.00	0.003	[1.00, 1.00]	12	0.80	[28.36, 99.49]	0.86	[42.13, 99.64]
Physician SMD-DT	-	-	-	12	0.80	[28.36, 99.49]	0.86	[42.13, 99.64]

Interrater agreement and diagnostic performance of different health professionals compared with the reference standard (Physician SMD-DT) in two phases across four clinical settings.

SMD-DT = spastic movement disorders decision tree, Cohens ϰ = Cohens Kappa.

Given the comparatively low agreement among nursing staff in the geriatric setting, the SMD-DT was evaluated in a neurological rehabilitation unit (A). In this setting, agreement levels were markedly higher. Both physiotherapists and nurses achieved high agreement with the reference standard (κ = 1.00; 95% CI: 1.00–1.00; *p* = 0.003), while occupational therapists showed substantial agreement (κ = 0.79; 95% CI: 0.00–1.00; *p* = 0.024). Sensitivity ranged from 0.60 for occupational therapists to 0.80 for physiotherapists and nurses, whereas specificity reached 1.00 for physiotherapists and occupational therapists and 0.86 for nurses (Table 3).

### 3.2 Phase 2

In phase 2, the newly developed SNQ (Table 1), applied by nursing staff, demonstrated substantial agreement, considering a recommendation for a referral, with the SMD-DT used by the physician (κ = 0.61; 95% CI: 0.00–1.00; *p* = 0.006). Sensitivity reached 1.00, indicating that all positive cases identified by the reference standard were later recommended for injection, while specificity was high (0.96), with only one false positive as shown in Table 4.

**Table 4:** Phase 2.

Phase 2								
Neurological rehabilitation Unit B								
vs. Physician SMD-DT	Cohen’s ϰ	*ρ*-value	95% CI	*n*	sensitivity	95% CI	specificity	95% CI
Salzburg Nursing Questionnaire	0.61	0.006	[0.00, 1.00]	28	1.00	[47.82, 100.00]	0.96	[78.05, 99.89]
Arzt SMD-DT	-	-	-	28	0.60	[14.66, 94.73]	1.00	[85.18, 100.00]

Long-term care facility					
	*n*	sensitivity	95% CI	specificity	95% CI
Salzburg Nursing Questionnaire	28	1.00	[79.41, 100.00]	1.00	[73.54, 100.00]

Interrater agreement and diagnostic performance of different health professionals compared with the reference standard (Physician SMD-DT) in two phases across four clinical settings.

SMD-DT = Spastic Movement Disorders decision tree^14^, Cohens ϰ = Cohens Kappa.

To minimize the likelihood of chance findings, the SNQ was additionally evaluated in an independent long-term care facility with a neurological focus. In this setting, the instrument showed high diagnostic performance when compared with the physician’s clinical recommendation for treatment with botulinum toxin, achieving both sensitivity and specificity of 1.00 (Table 4).

Overall, the SNQ demonstrated high diagnostic accuracy across both settings, with particularly strong performance in relation to physician recommendations for treatment with botulinum toxin in the long-term care facility and substantial agreement with SMD-DT in relation to referral recommendation in neurological rehabilitation settings.

## 4 Discussion

Optimal treatment for focal spasticity – or more broadly, focal SMD – remains challenging. In clinical practice, SMD rarely presents as an isolated phenomenon but rather as part of a complex constellation of upper motor neuron signs, including paresis, impaired selective motor control, involuntary muscle activity and secondary biomechanical changes. Accurate identification and interpretation within interprofessional and multidisciplinary care pathways therefore require substantial clinical expertise, particularly when distinguishing clinically relevant spasticity from other components of post-stroke motor impairment (14, 19, 20). These practical challenges are closely linked with the absence of a universally accepted definition of spasticity. Classical neurophysiological models capture only limited aspects of the disorder and fail to adequately reflect its functional and activity-related manifestations. More recent frameworks conceptualize SMD as a multidimensional sensorimotor disorder, which may partly explain the variability in its recognition and management across clinical disciplines (19, 21). This conceptual and operational heterogeneity likely contributes to the well-documented discrepancy between the epidemiological prevalence of spasticity and its treatment in routine care (8, 19, 22). Here structured, context-adapted screening approaches may facilitate referral for interventions such as botulinum toxin therapy and thereby improve functional outcomes while reducing long-term disability (2, 5–7).

Complex concepts for the detection and management of spasticity are difficult to implement in routine clinical care, particularly in non-specialized settings and may therefore contribute to underdiagnosis or delayed identification. This challenge is especially relevant in multidisciplinary care contexts, where healthcare professionals with diverse educational backgrounds, varying levels of experience in neurological rehabilitation and differing clinical priorities collaborate. Only recently has this issue received increased attention in the literature, with structured decision-support systems being proposed to standardize and facilitate the detection of spasticity (8, 20, 22). By enhancing clinical awareness and directing attention to relevant warning signs, such tools may support timely referral and interprofessional communication (1). The SMD-DT represents one such approach, integrating established clinical scales into an algorithmic framework. However, the present findings indicate that its performance is not consistent across all clinical contexts and professional groups. In the geriatric setting, agreement with the physician-based reference standard was limited among nursing staff, whereas substantially higher agreement was observed in neurological rehabilitation. All comparisons were statistically significant. However, confidence intervals were wider in the neurological rehabilitation unit, likely reflecting smaller sample sizes. Overall, interrater agreement and diagnostic accuracy were higher in the neurological rehabilitation setting compared with the geriatric ward. These differences likely reflect not intrinsic limitations of the instrument itself but rather contextual factors, including profession-specific training, routine exposure to SMD and familiarity with structured motor assessments. In this context, the SMD-DT demonstrated consistently strong performance when applied by physiotherapists and occupational therapists, particularly in the rehabilitation setting, where agreement reached substantial high levels. In contrast, its applicability in routine nursing practice appeared limited despite prior training. This likely reflects a mismatch between the requirements of the instrument – particularly the use of formal scales such as the MAS (11) and the MRC scale (12) – and the realities of nursing workflows outside of neurological rehabilitation. In such settings, these assessments are often only partially integrated into routine nursing practice, frequently due to time constraints and limited familiarity with their application (22, 23).

To bridge this gap, we propose the SNQ as a simplified, context-adapted screening instrument. In phase 2, this tool demonstrated a robust performance across different settings and despite considerable heterogeneity within the study population, with high sensitivity and high specificity considering the accuracy in recognizing a candidate potentially benefiting from an additional botulinum toxin injection. Beyond diagnostic accuracy, early identification through such screening may facilitate care and comfort for patients, contribute to a reduction in caregiver burden and help to prevent spasticity-related complications. These findings have important implications for clinical implementation. Rather than assuming universal applicability of a single instrument, early detection of SMD appears to require profession-specific and context-adapted strategies. Algorithm-based tools may be particularly effective in therapy-oriented disciplines, whereas simplified, function-oriented screening approaches seem better suited for nursing staff to enable timely identification and referral.

From a broader movement disorders perspective, this study highlights a central challenge: translating complex pathophysiological concepts into feasible clinical workflows. Bridging this gap requires not only conceptual clarity but also implementation strategies that are aligned with the realities of multidisciplinary care.

### 4.1 Limitations

This was a single-centre study, which may limit the generalizability of the findings to other healthcare systems or less specialized care settings. But, in both SNQ-tested cohorts’ sensitivity and specificity was high confirming the usability in a rehabilitation unit and in a long-term care facility.

Sample sizes across the individual study phases were limited, resulting in wide confidence intervals for sensitivity, specificity and agreement measures. In particular, the small number of patients with an indication for botulinum toxin therapy increases the susceptibility of sensitivity estimates to variability driven by individual cases.

For the indication of botulinum toxin therapy, no clearly operationalized or fully objective criteria exist, in contrast to certain nursing assessments – such as the SNQ – which rely on clearly dichotomous decision parameters (for example “pain: yes or no”). In this context, it should also be mentioned that especially in long-term care, the indication for an injection with botulinum toxin – based on the residents’ most pressing issues – aligns with this dichotomization, due to the main focus in facilitating care and comfort, reducing caregiver burden and preventing spasticity-related complications. Therefore, this approach might tend to neglect persons across a broader clinical spectrum. Against this background, the chosen reference standard – based on clinical expertise and experience of the evaluating physician – represents a pragmatic and clinically highly relevant basis for decision-making. It allows for an adequate representation of complex, individualized therapeutic goals that would be insufficiently captured by purely standardized criteria. Nevertheless, the inherent subjectivity, especially considering one neurologist for the whole study, combined with this approach may contribute to inter-individual variability and may moderately influence estimates of performance parameters such as sensitivity and specificity. Despite implemented standardization efforts, a residual observer bias cannot be fully excluded, although it appears acceptable in light of clinical applicability.

In addition, the SMD-DT primarily focuses on the upper extremities, which results in less attention being given to impairments of the lower extremities. Consequently, patients with spinal cord injuries or syndromes with similar manifestations affecting only the lower extremities are overlooked. This issue is addressed in the SNQ considering its applicability independent of the affected body compartment.

The SNQ used in this study was developed as a practice-oriented addition of existing decision structures (specifically the SMD-DT) within an internal, interprofessional consensus process. This development was prompted by observations from the implementation of the SMD-DT, which indicated that its transferability to settings with nurses potentially without a specific neurological background may be limited and that it does not consistently achieve its intended guidance function in such contexts. In contrast, its applicability proved considerably more robust in specialized neurorehabilitation settings. The SNQ was therefore specifically designed to facilitate better adaptation to the requirements of nurses in non-specialized care environments and in consideration of their clinical focus.

Despite this conceptual advancement, the instrument has not yet undergone formal psychometric validation. In particular, assessments of construct validity, test–retest reliability and external validation in independent cohorts are still pending. For sustainable and widespread implementation, a systematic scientific evaluation of these measurement properties is therefore required.

Regardless of structured training prior to study initiation, it is likely that differences in professional experience and prior exposure to the clinical management of spasticity influenced diagnostic performance. In particular, the application of more complex assessments, as required by the SMD-DT, appears to demand a level of clinical routine and domain-specific familiarity that cannot be fully achieved through one-time or short-term training formats. This may have contributed to the observed variability, particularly in less specialized settings and underscores the importance of continuous, practice-oriented training and supervision concepts for the implementation of such instruments.

## 5 Conclusion

A profession-specific screening approach may improve early detection and treatment of spasticity and help close the referral gap. The present study demonstrates that the identification of clinically relevant SMD is strongly influenced by both the clinical context and the professional background of the assessor. The SMD-DT provides a reliable basis for referral decisions when applied by physiotherapists and occupational therapists, particularly in rehabilitation settings. However, its applicability in routine nursing care appears limited. In contrast, a simplified, context-adapted screening instrument – the SNQ – enables accurate and practicable identification by nurses of patients requiring specialist evaluation.

These findings support a differentiated implementation strategy in which complementary, profession-specific instruments are integrated into clinical workflows. Such an approach may enhance detection, strengthen interprofessional communication and facilitate timely access to targeted interventions, including botulinum toxin therapy.

## Data Availability

The raw data supporting the conclusions of this article will be made available by the authors, upon reasonable request and in line with the General Data Protection Regulation (GDPR).
