# Supplementary material for: Improving detection of candidates for botulinum toxin therapy in spasticity: an interprofessional screening approach
Source: Front Neurol. 2026 Oct 15;17:1939701. doi: 10.3389/fneur.2026.1939701 (PMC13716860; doi:10.3389/fneur.2026.1939701)
Supplement: Supplementary file 1 [file Data_Sheet_1.pdf]

Supplement 1 – German original version of the Salzburg Nursing Questionnaire

Pflegerische Indikation Spastik	
Benötige ich Kraft bei der Körperpflege aufgrund der Spastik?<br>z.B. Intimbereich, Achsel, Harnkatheterwechsel	O
Werden Schmerzen bei passiver Mobilisation wahrgenommen?<br>z.B. beim Ankleiden, bei der Körperpflege	O
Bestehen Hautverletzungen (Mazerationen/Ulzerationen) aufgrund der Spastik?<br>z.B. Handfläche, Ellenbogen, Achsel	O

Only the original German version of the assessment was used in this study.
