# Supplementary material for: Improving detection of candidates for botulinum toxin therapy in spasticity: an interprofessional screening approach
Source: Front Neurol. 2026 Oct 15;17:1939701. doi: 10.3389/fneur.2026.1939701 (PMC13716860; doi:10.3389/fneur.2026.1939701)
Supplement: Supplementary file 2 [file Data_Sheet_2.pdf]

Supplement 2 – Phase 1 – 2x2 for Sensitivity and Specificity included

**Phase 1**									
**Geriatric ward**									
	**True positive**	**False positive**	**True negative**	**False negative**	**n**	**sensitivity**	**95% CI**	**specificity**	**95% CI**
Physiotherapist	2	3	48	1	54	0.67	[9.43, 99.16]	0.94	[83.76, 98.77]
Occupational therapist	2	1	52	1	56	0.67	[9.43, 99.16]	0.98	[89.93, 99.95]
Nurse SMD-DT	2	7	46	1	56	0.67	[9.43, 99.16]	0.87	[74.66, 94.52]
Physician SMD-DT	3	0	53	0	56	1.00	[29.24, 100.00]	1.00	[93.28, 100.00]
**Neurological rehabilitation Unit A**									
	**True positive**	**False positive**	**True negative**	**False negative**	**n**	**sensitivity**	**95% CI**	**specificity**	**95% CI**
Physiotherapist	4	0	6	1	11	0.80	[28.36, 99.49]	1.00	[54.07, 100.00]
Occupational therapist	3	0	6	2	11	0.60	[14.66, 94.73]	1.00	[54.07, 100.00]
Nurse SMD-DT	4	1	6	1	12	0.80	[28.36, 99.49]	0.86	[42.13, 99.64]
Physician SMD-DT	4	1	6	1	12	0.80	[28.36, 99.49]	0.86	[42.13, 99.64]

Interrater agreement and diagnostic performance of different health professionals compared with the reference standard (Physician SMD-DT) in two phases across four clinical settings.
SMD-DT = Spastic Movement Disorders decision tree[14], Cohen´s $\varkappa$ = Cohen´s Kappa, ASE = asymptomatic standard error, TPR = True Positive Rate, TNR = True Negative Rate, FPR = False Positive Rate, FNR = False Negative Rate
