# Supplementary material for: Improving detection of candidates for botulinum toxin therapy in spasticity: an interprofessional screening approach
Source: Front Neurol. 2026 Oct 15;17:1939701. doi: 10.3389/fneur.2026.1939701 (PMC13716860; doi:10.3389/fneur.2026.1939701)
Supplement: Supplementary file 3 [file Data_Sheet_3.pdf]

Supplement 3 – Phase 2– 2x2 for Sensitivity and Specificity included

Phase 2									
**Neurological rehabilitation Unit B**									
	**True positive**	**False positive**	**True negative**	**False negative**	**n**	**sensitivity**	**95% CI**	**specificity**	**95% CI**
Salzburg Nursing Questionnaire	5	1	22	0	28	1.00	[47.82, 100.00]	0.96	[78.05, 99.89]
Arzt SMD-DT	3	0	23	2	28	0.60	[14.66, 94.73]	1.00	[85.18, 100.00]
**Long-term care facility**									
	**True positive**	**False positive**	**True negative**	**False negative**	**n**	**sensitivity**	**95% CI**	**specificity**	**95% CI**
Salzburg Nursing Questionnaire	16	0	12	0	28	1.00	[79.41, 100.00]	1.00	[73.54, 100.00]

Interrater agreement and diagnostic performance of different health professionals compared with the reference standard (Physician SMD-DT) in two phases across four clinical settings.
SMD-DT = Spastic Movement Disorders decision tree[14], Cohen´s $\varkappa$ = Cohen´s Kappa, ASE = asymptomatic standard error, TPR = True Positive Rate, TNR = True Negative Rate, FPR = False Positive Rate, FNR = False Negative Rate
